# GSTP1 DNA Methylation and Expression Status Is Indicative of 5-aza-2′-Deoxycytidine Efficacy in Human Prostate Cancer Cells

**DOI:** 10.1371/journal.pone.0025634

**Published:** 2011-09-28

**Authors:** Karen Chiam, Margaret M. Centenera, Lisa M. Butler, Wayne D. Tilley, Tina Bianco-Miotto

**Affiliations:** Dame Roma Mitchell Cancer Research Laboratories, Discipline of Medicine, University of Adelaide, Hanson Institute, Adelaide, Australia; Florida International University, United States of America

## Abstract

DNA methylation plays an important role in carcinogenesis and the reversibility of this epigenetic modification makes it a potential therapeutic target. To date, DNA methyltransferase inhibitors (DNMTi) have not demonstrated clinical efficacy in prostate cancer, with one of the major obstacles being the inability to monitor drug activity during the trial. Given the high frequency and specificity of *GSTP1* DNA methylation in prostate cancer, we investigated whether *GSTP1* is a useful marker of DNMTi treatment efficacy. LNCaP prostate cancer cells were treated with 5-aza-2′-deoxycytidine (5-aza-CdR) either with a single high dose (5–20 µM), every alternate day (0.1–10 µM) or daily (0.005–2.5 µM). A daily treatment regimen with 5-aza-CdR was optimal, with significant suppression of cell proliferation achieved with doses of 0.05 µM or greater (p<0.0001) and induction of cell death from 0.5 µM (p<0.0001). In contrast, treatment with a single high dose of 20 µM 5-aza-CdR inhibited cell proliferation but was not able to induce cell death. Demethylation of *GSTP1* was observed with doses of 5-aza-CdR that induced significant suppression of cell proliferation (≥0.05 µM). Re-expression of the GSTP1 protein was observed only at doses of 5-aza-CdR (≥0.5 µM) associated with induction of cell death. Treatment of LNCaP cells with a more stable DNMTi, Zebularine required at least a 100-fold higher dose (≥50 µM) to inhibit proliferation and was less potent in inducing cell death, which corresponded to a lack of GSTP1 protein re-expression. We have shown that *GSTP1* DNA methylation and protein expression status is correlated with DNMTi treatment response in prostate cancer cells. Since *GSTP1* is methylated in nearly all prostate cancers, our results warrant its testing as a marker of epigenetic therapy response in future clinical trials. We conclude that the DNA methylation and protein expression status of *GSTP1* are good indicators of DNMTi efficacy.

## Introduction

Prostate cancer is one of the most commonly diagnosed male cancers in Western countries. Current therapies for clinically localized disease include surgical removal of the prostate gland (prostatectomy) and/or radiotherapy with or without androgen deprivation therapy (ADT). Since the discovery, in the 1940s, that prostate cancer is dependent on the male sex hormones [1], initially castration and subsequently various forms of ADT, either alone or combined with androgen receptor (AR) antagonists, have been the main therapy for metastatic disease. After an initial variable duration of tumor regression, most metastatic prostate cancers progress to a “castration-resistant” stage that is unresponsive to ADT. Currently there are limited treatment options available for castration-resistant prostate cancer and consequently there is a serious need to develop new therapies.

It is well-established that epigenetic alterations are common events in carcinogenesis, including prostate cancer, which may lead to aberrant expression of critical genes such as tumor suppressors and oncogenes. Unlike DNA mutations, epigenetic alterations are chemically reversible by agents known as epigenetic inhibitors and are therefore potential therapeutic targets. Examples of epigenetic inhibitors that have shown success as therapeutic agents include the DNA methyltransferase inhibitors (DNMTi), 5-aza-cytidine (5-aza-CR or Vidaza) and its more potent analogue 5-aza-2′-deoxycytidine (5-aza-CdR or Decitabine). 5-aza-CR and 5-aza-CdR are nucleoside DNMTi developed initially as cancer chemotherapeutic agents that are currently being used for the treatment of myelodysplastic syndromes (MDS) [2]. The demethylating actions of 5-aza-CR and 5-aza-CdR rely on their ability to incorporate into replicating DNA and covalently bind to the DNMT1 enzyme in an irreversible manner, which leads to DNMT1 protein degradation [2], [3]. As DNMT1 is required to maintain DNA methylation during replication, the degradation of DNMT1 subsequently results in a loss of DNA methylation.

Aberrant expression of epigenetic modifying enzymes involved in the regulation of DNA methylation has been observed at all stages of prostate cancer progression [4], [5], [6]. Global levels of 5-methylcytosine and epigenetic modifying enzymes involved in DNA methylation (i.e DNMTs) predict the likelihood of disease progression in prostate cancer. This finding suggests that DNA methylation may be important in progression of prostate cancer and therefore DNMTi should be considered as a potential treatment option [7], [8], [9]. While *in vitro* experiments and animal models have shown that 5-aza-CdR has anti-tumor activities in several cancers including prostate cancer [10], [11], [12], [13], [14], clinical trials of 5-aza-CdR for the treatment of solid tumors have not been successful due to drug related adverse events such as myelosuppression, nausea and vomiting [15], [16], [17]. In addition to toxicity issues, the efficiency of delivery and uptake of 5-aza-CdR to the tumor tissues, there is uncertainty about the optimal dose-schedule for specific tumor types [18]. To date, only one small phase II study with 5-aza-CdR in prostate cancer has been published, approximately a decade ago [16]. While there are ongoing clinical trials for 5-aza-CdR in various solid tumors, none of these trials are cancer-specific nor do they include prostate cancer (National Institutes of Health, US, clinicaltrials.gov). *In vitro* studies investigating the effects of 5-aza-CdR in prostate cancer cell lines (see Table S1) have used various treatment regimes and different definitions for low and high 5-aza-CdR doses, making it difficult to compare between studies and define the optimal treatment regime, including dose-schedule, for prostate cancer. Surprisingly, very few of the *in vitro* studies (Table S1A) have investigated the effects of 5-aza-CdR on the proliferation and survival of prostate cancer cells, but rather have investigated the effect of 5-aza-CdR on gene expression in order to identify candidate epigenetically-regulated genes (Table S1B).

The aim of this study was to investigate the dose-dependent effects of 5-aza-CdR in prostate cancer cells with view to providing a basis for developing an optimal 5-aza-CdR treatment regime for prostate cancer. We also investigated the relative toxicity of 5-aza-CdR and Zebularine in LNCaP prostate cancer cells. Zebularine is a cytidine analogue that has similar functions to 5-aza-CdR as a demethylating agent, but is less toxic and has a more stable half life (∼508 hours at 37°C, pH 7) than 5-aza-CdR (12 hours at 37°C, pH 7) [19], [20]. Identification of a good marker of DNMTi efficacy for clinical trials, much like the measurement of serum PSA levels to monitor the efficacy of ADT [21], would have the potential to aid clinical management of prostate cancer patients treated with epigenetic therapies. To investigate the efficacy of 5-aza-CdR and Zebularine in prostate cancer cells, we examined DNA methylation and expression status of the glutathione-S-transferase P1 (*GSTP1*) gene. *GSTP1* is hypermethylated in nearly all human prostate cancers and its promoter DNA methylation level is able to differentiate between benign prostatic hyperplasia and different grades of prostate adenocarcinoma [6], [22], [23], [24], [25]. While current studies have focused on using *GSTP1* as a potential marker for the early detection of prostate cancer, we propose that assessing DNA methylation of the *GSTP1* promoter region, as well as expression of GSTP1, has the potential to be a useful tool for determining DNMTi efficacy in prostate cancer.

## Materials and Methods

### Measurement of cell viability

LNCaP and PC3 human prostate carcinoma cells (American Type Culture Collection, ATCC) were maintained in RPMI 1640 supplemented with 5% or 10% fetal bovine serum (FBS). 5-aza-CdR and Zebularine (Sigma, A3656 and Z4775) were dissolved in dimethylsulfoxide (DMSO) and Hank's buffered salt solution respectively. For the single and every alternate day treatment with 5-aza-CdR, cells were seeded in triplicate in 24-well plates at a density of 2.5×10^4^ cells per well in 1 mL of RPMI medium. For the 5-aza-CdR daily treatment and Zebularine treatment, cells were seeded in triplicate in 12-well plates at a density of 1×10^4^ cells per well in 1 mL of RPMI medium. Cells were allowed to attach for 241h or 48 h, once cell confluency was reached and then incubated with medium containing 5-aza-CdR at concentrations of 0–20 µM or Zebularine at concentrations of 50–1000 µM. For subsequent or additional treatments, fresh 5-aza-CdR or Zebularine diluted in media was added to the cells. Media containing the respective agents were freshly prepared from 10 mM 5-aza-CdR and 70 mM Zebularine stocks before each treatment. Cells were trypsinized and counted using a hemocytometer at the specified time-points after initiation of treatment and cell viability assessed by Trypan blue dye exclusion as previously described [26]. Data are expressed as the mean +/− SE of triplicate wells and are representative of at least two independent experiments.

### Immunoblotting

LNCaP cells were seeded in 6-well plates at a density of 2×10^4^ cells per well in 2 mL of RPMI medium containing 10% FBS. Cells were allowed to attach for 24 h before medium was replaced with medium containing treatments. Cells were lysed by adding radioimmunoprecipitation assay lysis buffer (10 mM Tris-HCL, 150 mM NaCl, 1 mM EDTA, 1% Triton X-100) containing mini-complete protease inhibitor pellets (Roche). Lysates (15–30 µg) were electrophoresed through 5% or 12% polyacrylamide gels, transferred to nitrocellulose membrane (Amersham Biosciences), and blocked in 5% non-fat milk powder in TBS containing 0.05% Tween20 overnight. Immunodetection was performed with the specific primary antibody diluted in 1% non-fat milk powder in TBS containing 0.05% Tween20. GSTP1 antibody (Chemicon, AB8902) was used at a dilution of 1∶5000 and overnight incubation at 4°C. Hsp90 antibody (Santa Cruz Biotechnology) was used at a dilution of 1∶1000 and 30 min incubation at room temperature. Horseradish peroxidase-conjugated anti-rabbit secondary antibody (DAKO, E0432) was used at a dilution of 1∶2000 and 30 min incubation at room temperature. Results were visualized on Hyperfilm (GE Healthcare) using enhanced chemiluminescence detection (GE Healthcare).

### DNA methylation analysis

After cell viability assessment, the remaining LNCaP cells were collected for genomic DNA extraction using TES (10 mM Tris-HCL at pH 8, 0.1 M NaCl, 1 mM EDTA) buffer, proteinase K and 20% SDS as described previously [27]. DNA (1–2 µg per sample) was bisulfite modified with the MethylEasy™ DNA Bisulphite Modification Kit (Human Genetic Signatures Pty Ltd) according to the manufacturer's protocol. A total volume of 25 µl or 50 µl PCR reaction mix was made up with 3–5 µl of the bisulfite modified DNA and 2.5 units of HotstarTaq DNA polymerase (Qiagen). *GSTP1* Methylation-Specific Polymerase chain reaction (MSP) [28] and COmbined Bisulfite Restriction Analysis (COBRA) [29] primers were purchased from GeneWorks (South Australia, Australia). *GSTP1* MSP primer sequences were as described previously [24] and all primer sequences used in this study are provided in Figure S1. The annealing temperatures for the respective primers were: 40 cycles at 64.3°C for methylated *GSTP1* MSP primers; 45 cycles at 61.6°C for unmethylated *GSTP1* MSP primers; 45 cycles at 56.8°C for *GSTP1* COBRA primers. PCR products from the *GSTP1* COBRA analyzes were digested with restriction enzymes BstUI and HhaI (New England BioLabs). PCR products were visualized by agarose gel electropheresis with the AlphaImager 2200 gel documentation system (San Leandro).

### Statistical analysis

One-way analysis of variance (ANOVA) with a post-hoc Dunnet's multiple comparison test was used to compare cell viability between treatments and the vehicle control when a single time-point was assessed. Two-way ANOVA with a post-hoc Bonferroni test was used to compare cell viability between treatments and the vehicle control when multiple time-points were assessed. Analyses were performed with the GraphPad Prism 5 software (GraphPad Software, Inc., CA USA) and statistical significance was set at p<0.05 (two-sided).

## Results

### Daily 5-aza-CdR treatment is required to induce optimal inhibition of proliferation and induction of cell death in LNCaP prostate cancer cells

**Figure 1:**
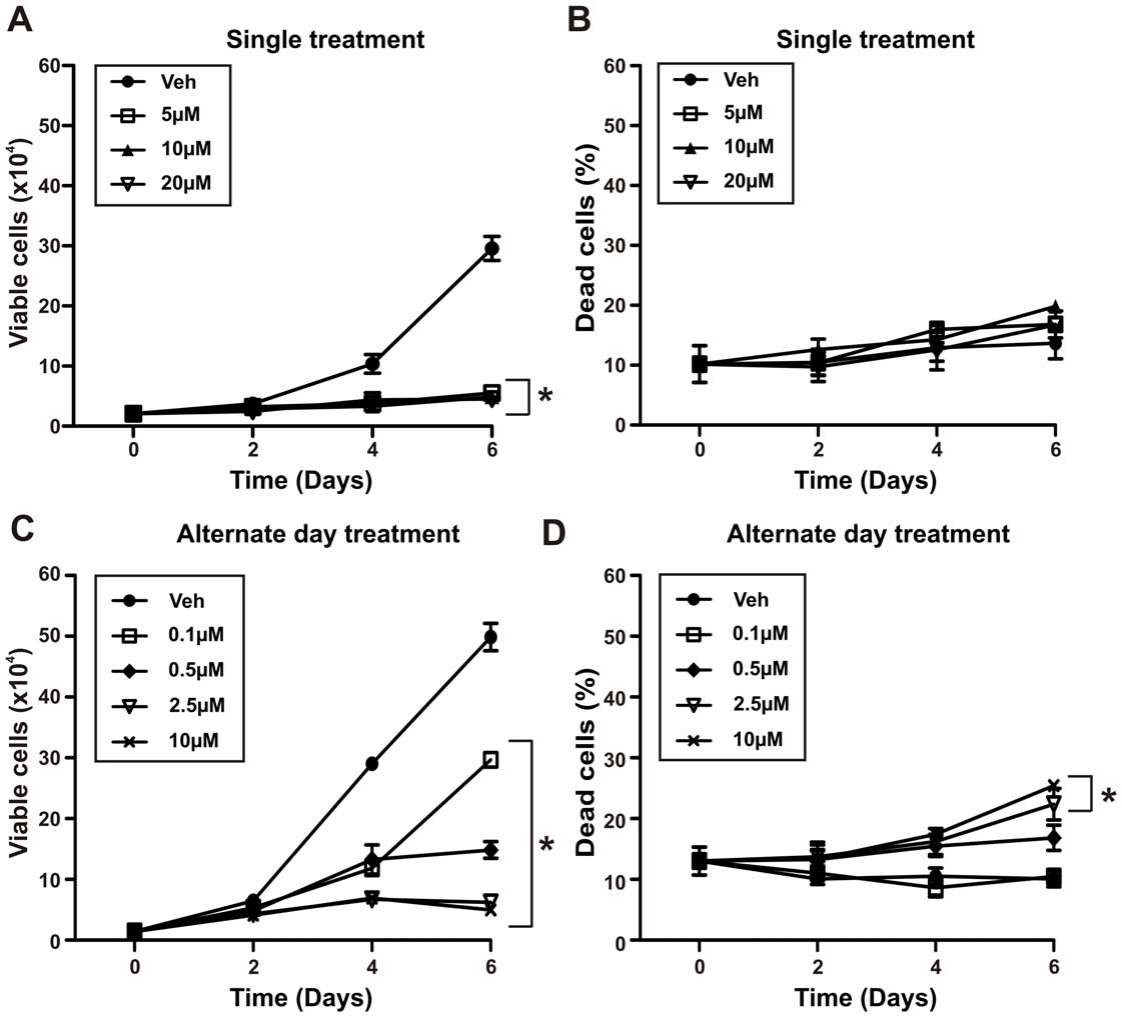
Single and alternate day 5-aza-CdR treatment of LNCaP prostate cancer cells. LNCaP prostate cancer cells (2.5×10^4^ cells per well in 24-well plates) were treated with increasing doses of 5-aza-CdR (5–20 µM) administered (A–B) once on day 0 or (C–D) with increasing doses of 5-aza-CdR (0.1-10 µM) replenished on alternate days for up to 6 days. (A) and (C) cells were counted at regular intervals using a hemocytometer and the number of viable cells was assessed by Trypan blue dye exclusion. (B) and (D) the number of dead cells is expressed as a percentage of the total number of cells counted. Data at each time-point represents the mean +/− SE of triplicate wells. *Two-way ANOVA: p<0.0001 for (A), (C) and (D) (10 µM); p<0.001 for (D) (2.5 µM) when compared to vehicle control (veh) on last day of treatment.

**Figure 2:**
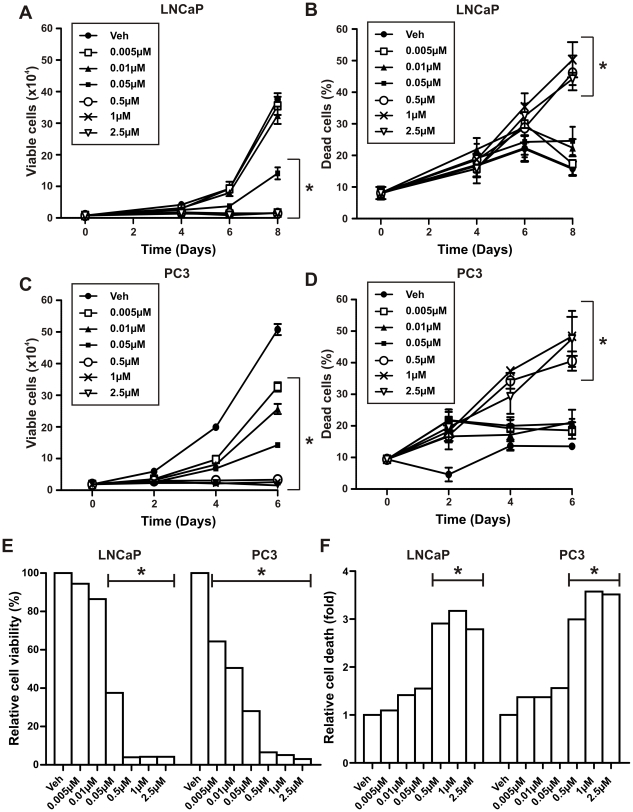
Daily 5-aza-CdR treatment of LNCaP and PC3 prostate cancer cells. (A–B) LNCaP and (C–D) PC3 prostate cancer cells (1×10^4^ cells per well in 12-well plates) were treated with increasing doses of 5-aza-CdR (0.005–2.5 µM) replenished daily for up to 8 days. (A) and (C) cells were counted at regular intervals using a hemocytometer and the number of viable cells was assessed by Trypan blue dye exclusion. (B) and (D) the number of dead cells is expressed as a percentage of the total number of cells counted. (E) and (F) relative cell viability following 6 or 8 days of treatment with 5-aza-CdR was presented as the percentage of viable cells compared to vehicle control (veh) and relative cell death as the fold of percent of dead cells compared to the veh control. Data at each time-point represents the mean +/− SE of triplicate wells from at least two experiments. *Two-way ANOVA: p<0.05 for (A) (0.01 µM); p<0.001 for (A) (0.05–2.5 µM), (B) and (D) (0.5 µM); p<0.0001 for (C) and (D) (1, 2.5 µM) when compared to vehicle control (veh) on last day of treatment.

To investigate the efficacy of different 5-aza-CdR treatment schedules, we performed cell proliferation and viability assays on LNCaP prostate cancer cells and compared the following: a single treatment, alternate day treatments and daily treatments. When compared to the control (vehicle), a single treatment of 5-aza-CdR effectively suppressed LNCaP prostate cancer cell proliferation at all concentrations used (5-20 µM) (Figure 1A, Day 4: p<0.001 for 5 µM and p<0.0001 for 10, 20 µM, Day 6: p<0.0001 for all doses) but did not induce significant cell death 6 days after treatment (Figure 1B). When 5-aza-CdR was added every second day (alternate day treatment), lower doses of 5-aza-CdR (0.1, 0.5 and 2.5 µM) compared to doses used in the single treatment schedule resulted in a significant dose-dependent inhibition of cell proliferation when compared to vehicle treated LNCaP cells (Figure 1C, Day 4 and 6: p<0.0001 for all doses). Only doses of 5-aza-CdR of 2.5 µM or greater induced significant cell death when compared to that of vehicle treated cells (Figure 1D, Day 6: p<0.001 for 2.5 µM and p<0.0001 for 10 µM). In contrast, daily treatment of 5-aza-CdR achieved significant inhibition of proliferation at lower concentrations (0.05 µM) (Figure 2A, Day 6: p<0.05 for 0.05 µM, p<0.001 for all doses 0.5 µM or greater, Day 8: p<0.05 for 0.01 µM and p<0.001 for all doses 0.05 µM or greater) and increased cell death in LNCaP cells (Figure 2B, Day 8: p<0.001 for doses 0.5 µM or greater) compared to similar doses given every second day. Dose-dependent inhibition of proliferation was achieved with 5-aza-CdR daily doses of 0.05 µM or greater resulting in a 62% reduction in cell number when compared to vehicle treated cells and complete inhibition of proliferation at doses of 0.5 µM or greater (Figure 2A and 2E, p<0.0001). At the doses that caused complete inhibition of proliferation, there was also a significant increase in cell death, of approximately 3-fold, when compared to vehicle control (Figure 2B and 2F, p<0.0001).

### Effects of 5-aza-CdR on prostate cancer cell viability is independent of the AR

To determine if the effects of 5-aza-CdR in LNCaP cells were dependent on a functional AR, a daily treatment schedule, was also performed in PC3 cells, which lack a functional AR. When treated with 5-aza-CdR at doses of 0.005–2.5 µM, there was a similar dose-dependent inhibition of proliferation and induction of cell death in PC3 cells as there was in LNCaP cells (Figure 2A–D). Whereas an approximate 3-fold induction of cell death was seen with 0.5 µM 5-aza-CdR in both cell lines (Figure 2F), in the PC3 cells, lower doses of 5-aza-CdR (0.005 µM and 0.01 µM) resulted in a significant reduction in cell number (p<0.0001, Figure 2E), and this occurred at an earlier time-point (4 days) when compared to LNCaP cells (6 days) treated identically (Figure 2A and 2C). Since 5-aza-CdR relies on dividing cells for incorporation to elicit its effects, the difference in doubling time between the 2 cell lines, approximately 24 hours in PC3 cells compared to 48 hours in the LNCaP cells, may explain the increased potency of 5-aza-CdR on PC3 cell viability. The androgen-independent inhibition of proliferation and induction of cell death by 5-aza-CdR in prostate cancer cells was further confirmed by cell viability assays performed in LNCaP cells cultured in steroid-depleted medium (Figure S2).

### Prolonged 5-aza-CdR treatment results in similar cell death regardless of the treatment regime

**Figure 3:**
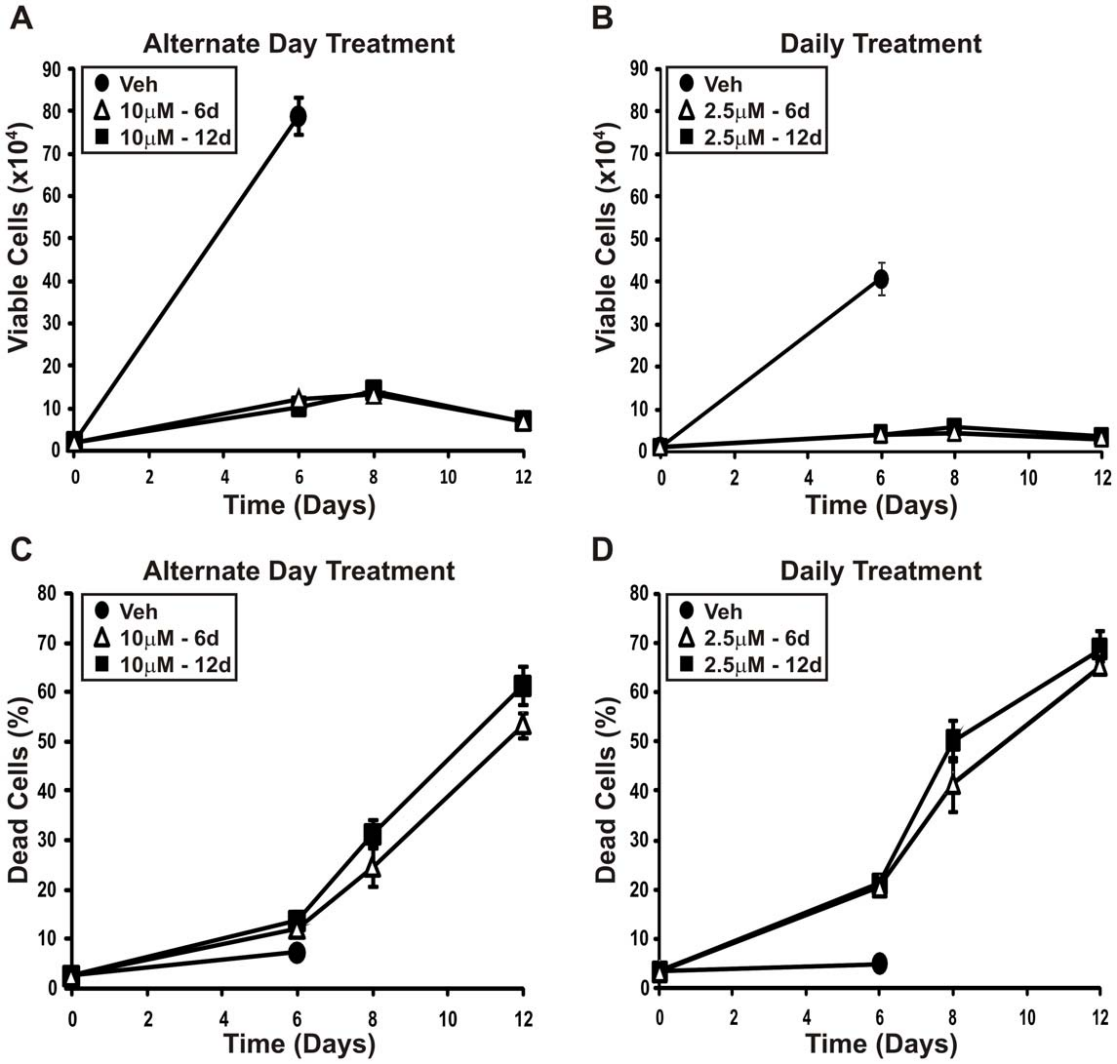
Prolonged alternate day or daily treatment with 5-aza-CdR in LNCaP cells results in similar cell death. (A) and (C) LNCaP prostate cancer cells (2.5×10^4^ cells per well in 24-well plates) were treated with 10 µM 5-aza-CdR or vehicle control, replenished on alternate days. (B) and (D) LNCaP prostate cancer cells (1×10^4^ cells per well in 12-well plates) were treated with 2.5 µM 5-aza-CdR or vehicle control, replenished daily. Following 6 days of treatment, control cells were ceased and the remaining cells either continued to receive 5-aza-CdR (10 µM-12d or 2.5 µM-12d, respectively) or received fresh media containing vehicle (10 µM-6d or 2.5 µM-6d). (A) and (C) cells were counted at day 6, 8 and 12 using a hemocytometer and the number of viable cells was assessed by Trypan blue dye exclusion. (B) and (D) the number of dead cells is expressed as a percentage of the total number of cells counted.

To further characterize the differences between the alternate day and daily treatment regime, the highest alternate day treatment (10 µM) and the highest daily treatment (2.5 µM) were compared in an extended growth curve (Figure 3). LNCaP prostate cancer cells were treated with vehicle control or 5-aza-CdR replenished on alternate days or daily, as above. Cell viability and cell death were assessed after 6 days of treatment. One set of cells then continued to receive 5-aza-CdR replenished on alternate days or daily until day 12 (denoted as 10 µM-12d or 2.5 µM-12d; Figure 3) while the other set of cells received media containing vehicle control (denoted as 10 µM-6d or 2.5 µM-6d; Figure 3). After 6 days of treatment, both the alternate day and daily treatment regimes induced growth suppression compared with vehicle control but only the daily treatment resulted in cell death (Figure 3). As the control cells had reached confluency by day 6, these cells were excluded for the remainder of the experiment. With continued treatment on either regime the amount of cell death continued to increase for the 12 days, reaching 61.4% for the alternate day treatment and 68.6% for the daily treatment. Interestingly, the cells that only received treatment for 6 days displayed equivalent levels of cell death to those that received treatment for 12 days (Figure 3).

### *GSTP1* promoter DNA methylation status and protein re-expression as markers of 5-aza-CdR efficacy

**Figure 4:**
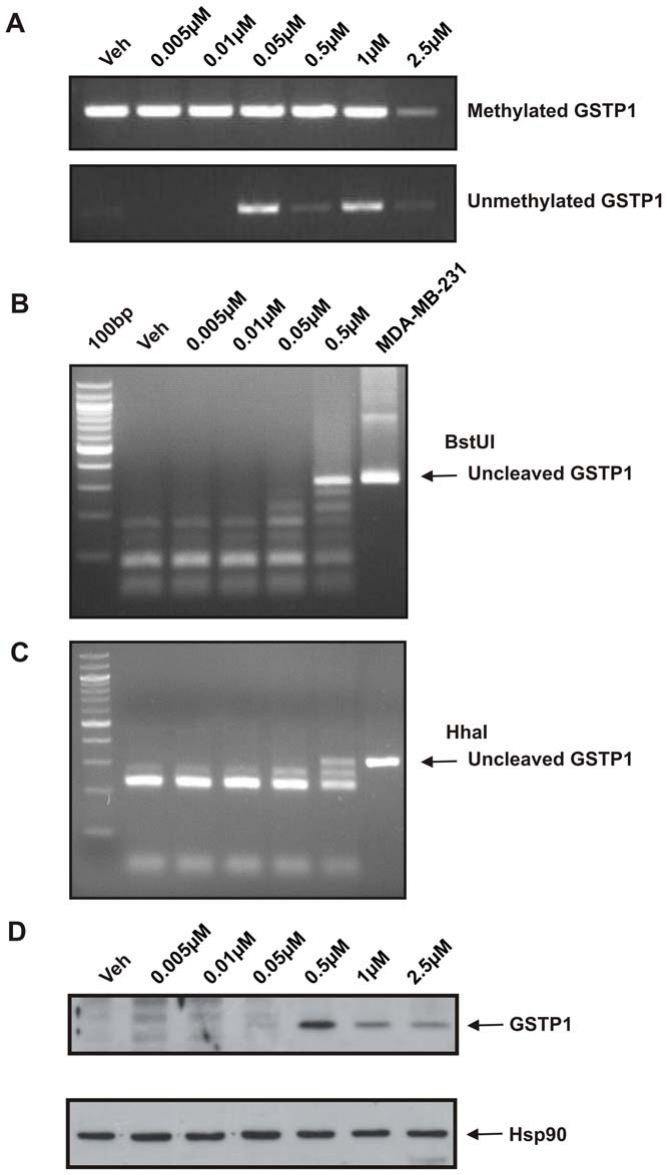
GSTP1 DNA methylation and protein expression in LNCaP cells after daily 5-aza-CdR treatment. DNA and proteins were extracted from LNCaP cells treated with increasing doses of 5-aza-CdR (0.005–2.5 µM). Cells were treated daily and DNA and protein harvested after 6 days of treatment. (A) MSP was performed on bisulfite-modified DNA with primers targeting bisulfite-modified methylated *GSTP1* promoter or unmethylated *GSTP1* promoter. (B–C) the relative methylation status of the GSTP1 promoter following 5-aza-CdR treatment was further assessed by COBRA using two restriction enzymes, BstUI and HhaI. MDA-MB-231 breast cancer cells were used as a control for unmethylated *GSTP1* promoter. (D) Immunoblot was performed to analyze GSTP1 protein expression. Detection of Hsp90 was used as a loading control.

To investigate how the anti-proliferative effects of 5-aza-CdR relate to its demethylating activity, MSP was performed to assess the DNA methylation status of the *GSTP1* promoter (Figure 4A). Hypermethylated *GSTP1* promoter DNA was present in LNCaP cells treated with vehicle control. In contrast, unmethylated *GSTP1* promoter DNA was detected in LNCaP cells treated daily with 0.05 µM or greater 5-aza-CdR, but completely demethylated *GSTP1* promoter DNA was not observed with even the highest concentration of 5-aza-CdR. Demethylation of the *GSTP1* promoter by 5-aza-CdR at doses great than or equal to 0.05 µM coincided with the ability of 5-aza-CdR to substantially inhibit cell proliferation at these concentrations (Figure 2A-B).

To further examine the relative DNA methylation status of the *GSTP1* promoter in the 5-aza-CdR treated LNCaP cells, COBRA was performed using BstUI and HhaI restriction enzymes (Figure 4B–C). The unmethylated *GSTP1* promoter present in MDA-MB-231 breast cancer cells was not digested by BstUI or HhaI (Figure 4B). Consistent with the MSP results, methylated *GSTP1* promoter was detected in vehicle treated (control) and 5-aza-CdR treated LNCaP cells at doses of 0.005–0.5 µM. Considerable *GSTP1* promoter DNA demethylation was only seen in response to 0.5 µM 5-aza-CdR, which is the lowest 5-aza-CdR dose sufficient to induce complete inhibition of LNCaP cell proliferation and cell death demonstrated in the cell viability assays (Figure 2A–B). These findings suggest that the efficacy of 0.5 µM 5-aza-CdR is due to its ability to induce greater DNA demethylation of *GSTP1* compared to lower doses.

The greater demethylating effect of 0.5 µM compared to 0.05 µM 5-aza-CdR corresponds with GSTP1 protein re-expression observed at 0.5 µM 5-aza-CdR or greater (Figure 4D). Consistent with this, the 5-aza-CdR doses that result in re-expression of GSTP1 protein also induce significant cell death in LNCaP cells (Figure 2B and 2F).

### Zebularine inhibits proliferation of prostate cancer cells but has limited effects on cell death

**Figure 5:**
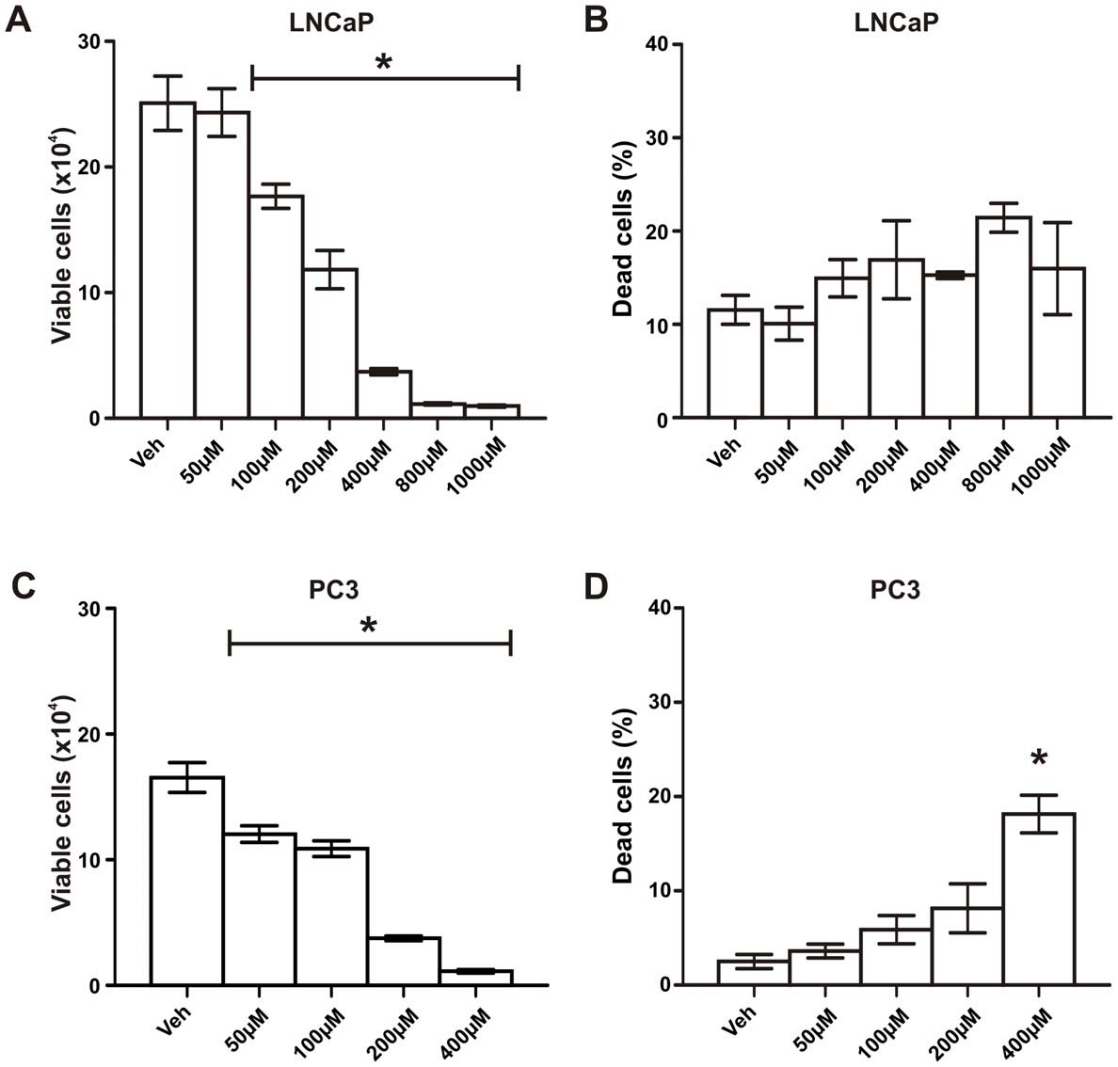
Effects of Zebularine treatment on LNCaP and PC3 prostate cancer cell viability and cell death. (A-B) LNCaP and (C–D) PC3 prostate cancer cells (1×10^4^ cells per well in 12-well plates) were treated with increasing doses of Zebularine (0–400 µM, up to 1000 µM for LNCaP cells) replenished once on day 4 for a period of 6 days for PC3 cells, and 8 days for LNCaP cells. (A) and (C) cells were counted at regular intervals using a hemocytometer and cell viability was assessed by Trypan blue dye exclusion. (B) and (D) the number of dead cells is expressed as a percentage of the total number of cells counted. Data at each time-point represents the mean +/− SE of triplicate wells. *One-way ANOVA; p<0.0001 for (A) and (C); p = 0.0004 for (D) compared to vehicle control (veh).

LNCaP cells were treated with Zebularine (0–1000 µM; highest dose used in previous studies [30]), while PC3 cells were treated with Zebularine doses of up to 400 µM. Zebularine was given on day 0 and replenished again halfway through the treatment period. Zebularine caused a dose-dependent inhibition of proliferation in both LNCaP and PC3 prostate cancer cells (Figure 5A and 5C, p<0.0001), suggesting that Zebularine has a similar AR-independent growth inhibitory mechanism of action on prostate cancer cells as 5-aza-CdR. A significant reduction in the number of viable cells was observed with 100 to 200 µM Zebularine, and complete inhibition of cell proliferation was observed at 400 µM or greater in both LNCaP and PC3 cells (Figure 5A and 5C, p<0.0001). Whereas Zebularine failed to induce cell death at any dose in LNCaP cells (Figure 5B), significant cell death was induced by 400 µM Zebularine in PC3 cells (Figure 5D, p = 0.0004).

### Zebularine has weaker demethylating actions on the *GSTP1* promoter compared to 5-aza-CdR

**Figure 6:**
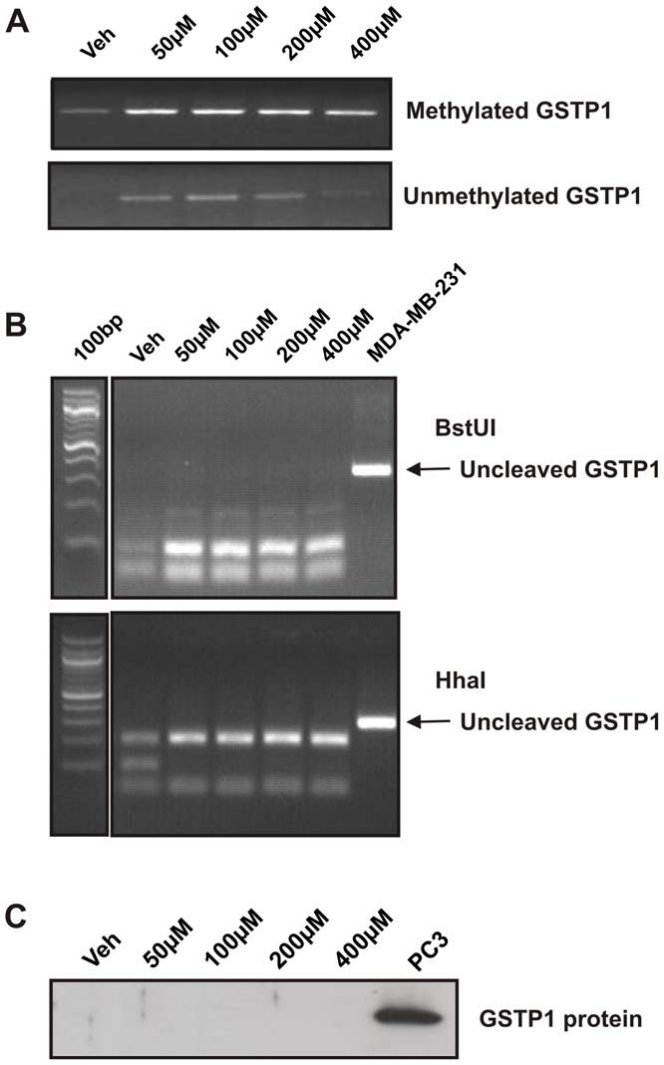
DNA methylation status and protein expression of *GSTP1* in LNCaP cells after Zebularine treatment. DNA and proteins were extracted from LNCaP cells after 8 days of treatment with increasing doses of Zebularine (0–400 µM). (A) DNA was bisulfite-modified and MSP was performed with primers targeting bisulfite-modified methylated *GSTP1* or unmethylated *GSTP1*. (B) The relative DNA methylation status of the *GSTP1* promoter following Zebularine treatment was assessed by COBRA using two restriction enzymes, BstUI or HhaI. (C) Immunoblot was performed to analyse GSTP1 protein expression in LNCaP cells after 8 days of Zebularine treatment. Proteins were extracted from LNCaP cells treated with increasing doses of Zebularine (0–400 µM). PC3 cells express endogenous GSTP1 protein and were used as positive control.

To investigate the demethylating activity of Zebularine, MSP was performed to examine the DNA methylation status of the *GSTP1* promoter in LNCaP cells (Figure 6A). After 8 days of treatment, methylated *GSTP1* was present in the vehicle control and all Zebularine treated samples (0–400 µM), while demethylation of the *GSTP1* promoter lacked dose-dependency (Figure 6A). When the relative DNA methylation status of the *GSTP1* promoter region in these Zebularine-treated LNCaP cells were compared by COBRA using BstUI and HhaI restriction enzyme digestion, no unmethylated *GSTP1* was detected (Figure 6B). The weak and inconsistent demethylating actions of Zebularine on LNCaP cells was also reflected in the lack of GSTP1 protein re-expression after 8 days of treatment (Figure 6C).

## Discussion

While the DNMTi 5-aza-CdR is effective in the treatment of hematologic conditions, clinical trials in solid tumors and in prostate cancer have shown limited or no efficacy. The failure of previous clinical trials in solid tumors has been attributed to inappropriate dose regimens, leading to toxicity-related adverse events. In part, this is due to a poor understanding of the mechanistic actions of 5-aza-CdR in solid tumors. In this study, we demonstrate that 5-aza-CdR, at a dose of 0.5 µM given daily, completely inhibited cell proliferation and induced cell death in prostate cancer cells, and was associated with demethylation of the *GSTP1* promoter and re-expression of GSTP1 protein. These findings suggest that a daily low-dose 5-aza-CdR treatment regimen may be more effective than a less frequent or single high-dose schedule for the control of prostate cancer cell growth. We have also demonstrated that a daily low-dose 5–aza-CdR treatment regimen is more effective than one using the more stable DNMTi, Zebularine. Most importantly, we provide evidence that the increased potency of 5-aza-CdR compared to Zebularine in prostate cancer cells is closely related to its demethylating activity and identified *GSTP1* as a potentially useful biomarker for assessing DNMTi efficacy in prostate cancer.

Several studies have demonstrated that 5-aza-CdR reduces cell proliferation and induces re-expression of specific genes in various cancers (Table S1). Different cancer types respond to 5-aza-CdR differently, but a wide range of 5-aza-CdR doses and treatment regimens have been used in previous studies, and the end-points and analysis were different in the various studies. Nine published studies have investigated the effects of 5-aza-CdR on the viability of prostate cancer cell lines (Table S1A) [10], [11], [31], [32], [33], [34], [35], [36], [37]. Comparisons among these studies are difficult due to the reasons listed above. For instance, Walton et al [11] reported approximately 30% inhibition of cell proliferation compared to vehicle control in the LNCaP prostate cancer cells after treatment with 8.8 µM 5-aza-CdR, while Pulukuri et al [10] reported 70% inhibition of cell proliferation compared to vehicle control in the same cell line with a high dose of 10 µM 5-aza-CdR treatment. In contrast, our prolonged 5-aza-CdR daily treatment regime resulted in approximately 62% inhibition of cell proliferation in LNCaP cells treated with 0.05 µM 5-aza-CdR, a dose 200-fold lower than what was used by Pulukuri et al [10] to achieve a similar level of inhibition on cell proliferation. Therefore, it would appear that administration of a low daily dose of 5-aza-CdR is optimal for inhibition of prostate cancer cells by this DNMTi.

In our one-time high dose 5-aza-CdR treatment of prostate cancer cell lines, we chose a range of doses commonly used in previous studies and found that while these doses of 5-aza-CdR inhibited prostate cancer cell proliferation, they did not induce cell death. This is consistent with the study by Walton et al, where 5-aza-CdR failed to induce cytotoxicity in prostate cancer cell lines even at a very high dose of 100 µM [11]. However, by increasing the frequency of administration of 5-aza-CdR, we increased its efficacy such that previously ineffective low doses of 5-aza-CdR became sufficient to both inhibit cell proliferation and induce cell death. As 5-aza-CdR is degraded within 12 hours [19], [20], [30], it is not able to incorporate into replicating DNA to elicit its demethylating actions in the single treatment regime. Replenishment of 5-aza-CdR daily ensures that sufficient levels of the drug are sustained throughout the treatment period to improve efficacy. While previous studies have performed similar 5-aza-CdR daily treatments in other cancer cell lines, the treatment period (3–4 days only) was relatively short and did not achieve the same efficacy in terms of cell proliferation and cell death when compared to the treatment regimen used in our study [38], [39].

The rationale for a low dose daily 5-aza-CdR treatment regime for prostate cancer *in vitro* is similar to that for the prolonged low dose treatment used in hematological malignancies [40], [41], [42], [43]. The initial development of 5-aza-CdR as an anti-leukemic agent started when ‘pioneer’ studies demonstrated its efficacy in leukemic cell lines and mouse models [44], [45]. 5-aza-CdR was able to influence leukemic cell differentiation and induce gene expression that was associated with its DNA demethylating activity [2]. This led to initial clinical trials with 5-aza-CdR in patients with acute leukemia in the 1980s, branching later into clinical trials with several hematopoietic malignancies including myelodysplastic syndrome (MDS), sickle cell anemia and solid tumors [15], [46], [47]. However, results of these trials were not promising and were limited by poor pharmacokinetics, toxicity and an ineffective dose schedule. It was not until the late 1990s when a prolonged low dose schedule of 5-aza-CdR was introduced that promising results were achieved in clinical trials for the treatment of hematopoietic malignancies [40], [41], [42], [43]. The new dose schedule, based on an improved understanding of 5-aza-CdR mechanisms, was crucial for the development of 5-aza-CdR as a therapeutic agent. Other studies have shown that 5-aza-CdR is an S-phase specific agent, and that low and high doses of 5-aza-CdR have differential actions. Most importantly, low doses of 5-aza-CdR were sufficient to induce demethylation and re-expression of genes, without the cytotoxicity associated with higher doses [48], [49].

In addition to its demethylating activity, previous studies have shown that 5-aza-CdR is involved in several signaling pathways including cell cycle, DNA damage repair, apoptosis and angiogenesis [50]. For instance, 5-aza-CdR anti-tumor activities are p53-dependent [10], [51]. Studies by both Pulukuri et al and Karpf et al demonstrated that p53 positive cancer cell lines were more sensitive to 5-aza-CdR compared to p53 negative cell lines [10], [51]. We, however, observed similar 5-aza-CdR responses in the p53 positive LNCaP and p53 negative PC3 prostate cancer cells lines, suggesting that p53 independent mechanisms were invoked by the low dose daily treatment regime utilized in this study.

In this study, we also provide evidence that 5-aza-CdR does not require expression of a functional AR to elicit its effects in prostate cancer cells. The AR is critical for the maintenance of normal prostate function and the development and progression of prostate cancer, and is the main target in current treatments for prostate cancer. 5-aza-CdR anti-tumor activities were similar in LNCaP and PC3 cells, the latter lacking a functional AR. 5-aza-CdR remained effective in reducing cell viability in LNCaP cells in the absence of androgens, suggesting an androgen-independent mechanism. These findings are supported by *in vivo* studies of 5-aza-CdR in the TRAMP mouse model of prostate cancer [13], [14]. Upon castration, the TRAMP mouse develops “castration-resistant” prostate tumors similar to that seen with the recurrence of human prostate tumor growth after androgen-deprivation therapy. Treatment with 5-aza-CdR was found to increase survival of TRAMP mice and delayed prostate cancer progression, including the recurrence of prostate tumor growth after castration [13], [14]. Together, these results infer a potential role for epigenetic therapies such as 5-aza-CdR in the treatment of prostate cancer regardless of AR or androgen status.

While there remains controversy as to whether the anti-tumor activity of 5-aza-CdR is due to its demethylating activity or formation of DNA adducts [52], [53] one hypothesis is that low doses of 5-aza-CdR and high doses of 5-aza-CdR act via different mechanisms to elicit their anti-tumor effects. A major finding in this study is the correlation between 5-aza-CdR demethylation activity with inhibition of cell proliferation and GSTP1 protein re-expression and induction of cell death. Although past studies have shown that 5-aza-CdR was able to demethylate and re-express GSTP1 in prostate cancer cells [54], [55], [56], our results are the first to demonstrate that *GSTP1* methylation and protein status was indicative of 5-aza-CdR treatment efficacy using a daily low-dose treatment regime. These results support the hypothesis of a differential mechanism between “low” (inhibition of cell proliferation only) and “high” (induction of cell death) doses of 5-aza-CdR. Interestingly, the presence of GSTP1 protein itself does not influence prostate cancer cell proliferation [54], yet its DNA methylation and protein status seems to be indicative of the efficacy of DNMTi treatment. Furthermore, the DNA methylation and protein status of GSTP1 was indicative of the poor treatment response with Zebularine. Even though Zebularine effectively reduced prostate cancer cell number, it was unable to induce significant cell death, possibly due to its weak demethylating activity and inability to reactivate silenced genes such as *GSTP1*. Although initial studies suggested that Zebularine may be a better DNMTi than 5-aza-CdR for clinical use, this and other studies suggest that Zebularine is not as effective as 5-aza-CdR as a demethylating agent [39], [57].

One of the obstacles in previous clinical trials with DNMTis such as 5-aza-CdR, was the inability to investigate the efficacy of the drug in patients until the conclusion of the trial. Based on the findings of this study, we propose that *GSTP1* is a marker of DNMTi treatment efficacy in prostate cancer. The ability to track efficacy of the drug using tissue biopsies or circulating tumor cells at earlier time-points will greatly assist future clinical trials. Firstly, it has the potential to improve the assessment of drug efficacy, thus reducing both the duration and cost of a clinical trial, and secondly to improve the welfare of patients in clinical trials by minimizing unnecessary exposure. Another advantage of using *GSTP1* as a marker of DNMTi efficacy is that it can be easily measured in a patient's serum [58] or circulating tumor cells which will facilitate its use as a biomarker in future clinical trials. *GSTP1* status after neoadjuvant treatment with DNMTi may also be a useful prognostic marker, similar to the prognostic significance of Ki67 after neoadjuvant treatment with endocrine and chemo-therapies in breast cancer [59], [60].

## Supporting Information

Figure S1 ***GSTP1*** **COBRA and MSP primers specific for bisulfite modified unmethylated and methylated** ***GSTP1*** **.** The capital T defines thymines that are converted from cytosine residues by bisulfite modification. Unmethylated CpGs become TpG (**Tg**) and methylated CpGs **(cg**) remain unchanged upon conversion. The *GSTP1* COBRA primers were designed to target both unmethylated and methylated *GSTP1*. Following PCR amplification, PCR products were digested with either BstUI or HhaI restriction enzymes. The restriction sites identified by BstUI (CG_CG) are highlighted by **bold lines** while the restriction sites for HhaI (C_CGC) are highlighted by **dashed line**. The *GSTP1* MSP primers consist of one set of primers specific for unmethylated *GSTP1* and another set of primers specific for methylated *GSTP1*. The start site of *GSTP1* exon1 is indicated as +1.
(TIF)

Figure S2 **5-aza-CdR daily treatment of LNCaP and PC3 cells in steroid-depleted culture environment.** (A–B) LNCaP and (C–D) PC3 cells were cultured in steroid-depleted medium and treated with increasing doses of 5-aza-CdR (0.005–2.5 µM) replenished daily for a period of 8 or 6 days respectively. (A) and (C) cells were counted at regular intervals using a hemocytometer and cell viability was assessed by Trypan blue dye exclusion. (B) and (D) the number of dead cells is expressed as a percentage of the total number of cells counted. Data at each time-point represents the mean +/− SE of triplicate wells. *One-way ANOVA; p<0.0001 for (A) and (C); p = 0.007 for (D) compared to vehicle control (veh).
(TIF)

Table S1 **Summary of studies investigating 5-aza-cytidine (5-aza-CR) or 5-aza-2′-deocycytidine (5-aza-CdR) in prostate cancer cells.**
(DOCX)
