# Supplementary figures and images for: GSTP1 DNA Methylation and Expression Status Is Indicative of 5-aza-2′-Deoxycytidine Efficacy in Human Prostate Cancer Cells
Source: PLoS One. 2011 Sep 28;6(9):e25634. doi: 10.1371/journal.pone.0025634 (PMC3182253; doi:10.1371/journal.pone.0025634)

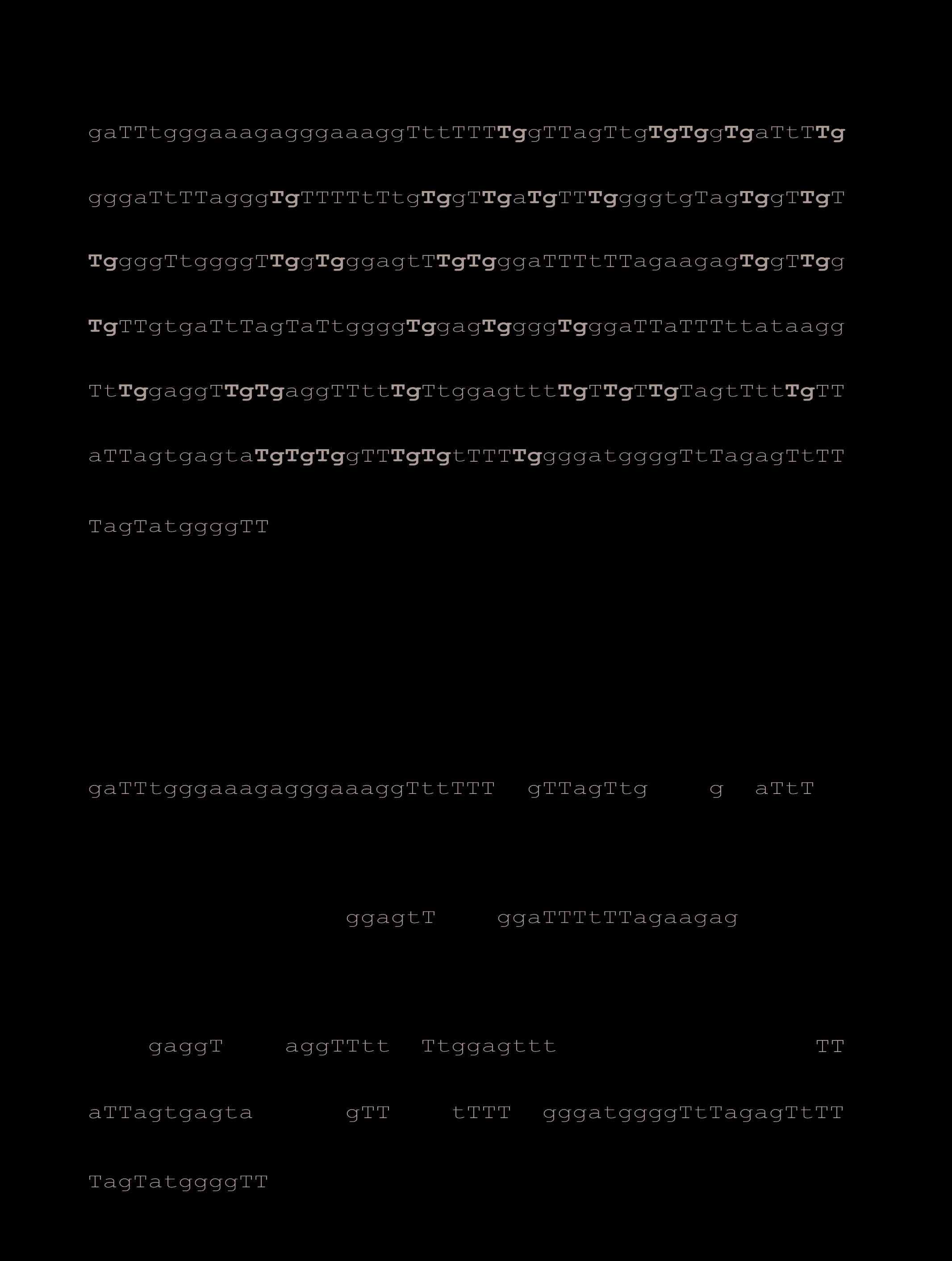

Supplement: Figure S1 — GSTP1 COBRA and MSP primers specific for bisulfite modified unmethylated and methylated GSTP1 . The capital T defines thymines that are converted from cytosine residues by bisulfite modification. Unmethylated CpGs become TpG (Tg) and methylated CpGs (cg) remain unchanged upon conversion. The GSTP1 COBRA primers were designed to target both unmethylated and methylated GSTP1. Following PCR amplification, PCR products were digested with either BstUI or HhaI restriction enzymes. The restriction sites identified by BstUI (CG_CG) are highlighted by bold lines while the restriction sites for HhaI (C_CGC) are highlighted by dashed line. The GSTP1 MSP primers consist of one set of primers specific for unmethylated GSTP1 and another set of primers specific for methylated GSTP1. The start site of GSTP1 exon1 is indicated as +1. (TIF) [file pone.0025634.s001.tif]

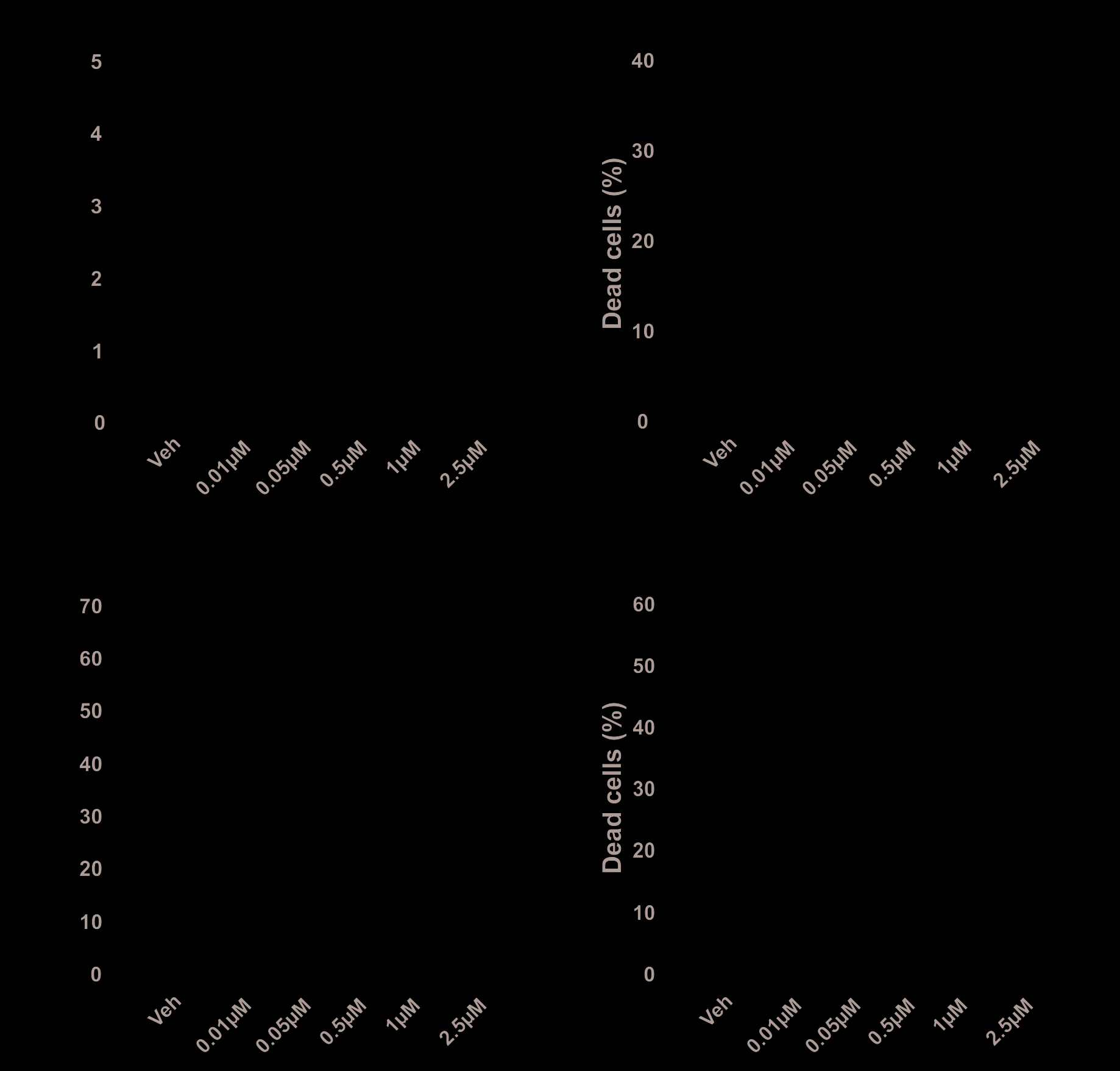

Supplement: Figure S2 — 5-aza-CdR daily treatment of LNCaP and PC3 cells in steroid-depleted culture environment. (A–B) LNCaP and (C–D) PC3 cells were cultured in steroid-depleted medium and treated with increasing doses of 5-aza-CdR (0.005–2.5 µM) replenished daily for a period of 8 or 6 days respectively. (A) and (C) cells were counted at regular intervals using a hemocytometer and cell viability was assessed by Trypan blue dye exclusion. (B) and (D) the number of dead cells is expressed as a percentage of the total number of cells counted. Data at each time-point represents the mean +/− SE of triplicate wells. *One-way ANOVA; p<0.0001 for (A) and (C); p = 0.007 for (D) compared to vehicle control (veh). (TIF) [file pone.0025634.s002.tif]
