# Supplementary material for: GSTP1 DNA Methylation and Expression Status Is Indicative of 5-aza-2′-Deoxycytidine Efficacy in Human Prostate Cancer Cells
Source: PLoS One. 2011 Sep 28;6(9):e25634. doi: 10.1371/journal.pone.0025634 (PMC3182253; doi:10.1371/journal.pone.0025634)
Supplement: Table S1 — Summary of studies investigating 5-aza-cytidine (5-aza-CR) or 5-aza-2′-deocycytidine (5-aza-CdR) in prostate cancer cells. (DOCX) [file pone.0025634.s003.docx]

**Table S1A: Summary of studies investigating effects of 5-aza-cytidine (5-aza-CR) or 5-aza-2’-deocycytidine (5-aza-CdR) on human prostate cancer cell viability**

| **Authors [Ref]** | **Cell lines** | **Treatment schedule** | **Outcome** |
| --- | --- | --- | --- |
| Gravina et al [1] | DU145, PC3 | 2.5, 5 or 10µM  5-aza-CR  daily treatment for 20 days | 5µM 5-aza-CR induced 50-60% growth suppression after 20 days  30% growth suppression by a high dose of 10µM 5-aza-CR after 4 days |
| Shang et al [2] | DU145, LNCaP, androgen-independent LNCaP, PC3 | 0.1, 1, 2, 4, 8 or 16µM  5-aza-CdR  one time treatment for 3 days | 16µM 5-aza-CdR induced 60% growth suppression, but no cell death (caspase 3 and 9 activities) was observed. |
| Walton et al [3] | DU145, LNCaP, PC3 | 0.1, 1, 8.8, 10 or 100µM  5-aza-CdR  one time treatment for 5 days | 5-aza-CdR treatment did not induce any significant cytotoxicity. 8.8µM 5-aza-CdR induced significant cell growth suppression, with a maximum 2-fold suppression in LNCaP cells. |
| Festuccia et al [4] | DU145,LNCaP, PC3, 22rv1 | 0.5-20µM  5-aza-CdR  24h treatment followed by drug free medium for another 3 days | 50% growth suppression with 0.5µM in 22rv1, 1µM in LNCaP, 5µM in PC3, 25µM in DU145. Dose- and time-dependent induction of apoptosis modulators in LNCaP and 22rv1 cells. |
| Richter et al [5] | LAPC4, LNCaP | 5 or 15µM  5-aza-CdR containing media changed every 2 days for 14 days | 15µM 5-aza-CdR induced 13-fold and 8-fold decrease in cell growth in LAPC4 and LNCaP cells respectively. |
| Bott et al [6] | DU145, LNCaP, PC3, 1542 NP | 0.5µM  5-aza-CdR  24h treatment followed by drug free medium for 5 days | 5-aza-CdR treatment significantly reduced cell proliferation rate by 45% in PC3, 40% in LNCaP and 15% in DU145. |
| Zhang et al [7] | LNCaP, RWPE-1 | 2µM  5-aza-CdR  Cells plated with normal media (day 0), followed by serum-free media on day 2, 3 and 5 days until day 7.5. | 50% cell growth suppression was observed in the LNCaP cells. |
| Pulukuri et al [8] | DU145, LNCaP | 0.01- 25µM  5-aza-CdR  one time treatment for 3 days | Maximum of 10µM 5-aza-CdR used in cell proliferation assays induced 70% and 40% cell growth suppression in LNCaP and DU145 respectively. |
| Hurtubise et al [9] | DU145 | 0.004- 4.4µM  5-aza-CdR  one time treatment for 2 days | 5-aza-CdR treatment induced a dose-dependent decrease in clonogenicity. |
| Kitagawa et al [10] | DU145, TSU-Pr1 | 1 or 5µM  5-aza-CR  replenished every 2 days for 5 days | 5µM 5-aza-CR induced approximately 50% growth suppression in both DU145 and TSU-Pr1 cells. No cell death was observed. |
| Steiner et al [11] | PPC-1 | 1µM  5-aza-CdR  daily treatment for 8 days | 5-aza-CdR treatment induced complete growth suppression in PPC-1 cells. |

**Table S1B: Summary of studies investigating 5-aza-cytidine (5-aza-CR) or 5-aza-2’-deocycytidine (5-aza-CdR) for epigenetic and gene expression analyzes in prostate cancer cells.**

| **Authors [Ref]** | **Cell lines** | **Treatment schedule** | **Genes of interest** |
| --- | --- | --- | --- |
| Chang et al [12] | DU145, LNCaP, PPC-1, TSU-pr1 | 2.5 or 5µM ; 5-aza-CdR  daily treatment for 4 days | CADM-2 |
| Ibragimova et al [13] | DU145, LNCaP, MDA2b, PC3 | 5µM ; 5-aza-CdR  daily treatment for 3 days | Identification of epigenetically silenced genes by microarrays |
| Kwabbi-Addo et al [14] | pNT1A, DU145, PC3, LNCaP | 5µM; 5-aza-CdR  one time treatment for 3 days | SPARC, TIMP3, NKX2.5 |
| Mϋller et al [15] | DU145, LNCaP | 1µM ; 5-aza-CdR  one time treatment for 3 days | CD44, CyclinD2, GLIPR, PTEN |
| Song et al [16] | DU145, PC3 | 10 or 25µM; 5-aza-CdR  one time treatment for 6 days | CXCL14 |
| Kwon et al [17] | 267B1, 267B1/K-ras | 10µM; 5-aza-CdR  one time treatment for 4 days | E-cadherin, K-ras |
| Schayek et al [18] | DU145, C4-2, PC3, P69, P69 transformed cells (M2205, M2182, M12) | 1µM; 5-aza-CdR  daily treatment for 3 days | AR, IGFR1 |
| Zhang et al  [19] | DU145 | 5µM; 5-aza-CdR  one time treatment for 7 days | Keap1 |
| Wu et al  [20] | DU145, LNCaP, LNCaP^CS^  (androgen-independent), PC3 | 0.5 or 0.75µM; 5-aza-CdR  new drug-containing media on day 4 for 8 days | PMP24 |
| Zhang et al  [21] | PC3 | 0.1, 0.2, 0.5, 2 or 5µM; 5-aza-CdR  new drug-containing media every 2 days for 5 days | PMP24 |
| Majid et al [22] | LNCaP, PC3 | 5µM; 5-aza-CdR  daily treatment for 5 days | BTG3 |
| Pandey et al [23] | LNCaP | 5, 10 or 20µM; 5-aza-CdR  one time treatment for 7 days | GSTP1 |
| Vanaja et al [24,25,26] | LAPC4, LNCaP, PC3 | 6µM; 5-aza-CdR  new drug-containing media on day 1, 3 and 5 for 6 days | ZNF185, PDLIM4, retinoic receptor-beta (RAR-beta) |
| Cheng et al [27] | PC3 | 2µM; 5-aza-CdR  daily treatment for 4 days | TNFRSF10C |
| Guan et al [28] | DU145 | 1 or 5µM; 5-aza-CdR  one time treatment for 4 days | EphA7 |
| Liu et al [29] | LNCaP, androgen-independent LNCaP | 2.5µM; 5-aza-CdR  new drug-containing media on day 3 for 5 days | Somatostatin receptor |
| Wang et al [30] | PC3 | 1, 3 or 10µM; 5-aza-CdR  one time treatment for 24 h | Endonuclease G |
| Liu et al [31] | DU145, PC3, 22rv1 | 5µM; 5-aza-CdR  daily treatment for 4 days | ssDNA-binding protein 2 |
| Chung et al [32] | PC3 | 1µM; 5-aza-CdR  daily treatment for 3 days | SPOCK2, NKX2.5 |
| Fujii et al [33] | LNCaP | 2µM; 5-aza-CdR  daily treatment for 2 days | RUNX3 |
| Menschikowski et al [34] | DU145 | 1, 5 or 10µM; 5-aza-CdR  one time treatment for 2-4 days | SPLA-IIA |
| Cangemi et al [35] | DU145, PC3 | 5-25µM; 5-aza-CdR  one time treatment for 2-4 days | Epithelial specific (ESE)-3 factor |
| Benbrahim-Tallaa et al [36] | Cadmium-induced malignant prostate epithelial cells | 0.5 or 1µM; 5-aza-CdR  replenished every 2 days for 6 days | RASSF1A |
| Dubovsky et al [37] | DU145, LNCaP,  RWPE-1, 22rv1 | 1, 5 or 10µM; 5-aza-CdR  one time treatment for 3 days, followed by drug-free media for 20 days | SSX-2 |
| Hoffmann et al [38,39] | DU145, PC3, LNCaP, 22rv1 | 2µM; 5-aza-CdR  daily treatment for 3 days | CTCFL, fibulin genes |
| Kawamoto et al [40] | LNCaP, PC3, RWPE-1, PWR-1E | 5µM; 5-aza-CdR  one time treatment for 3 days | RASSF1A |
| Hasegawa et al [41] | LNCaP, PC3 | 1µM; 5-aza-CdR  one time treatment for 5 days | RIZ1 |
| Bastian et al [42] | BPH-1, DU145, LNCaP, PC3 | 1µM; 5-aza-CdR  one time treatment for 3 days | PTGS2, RAR-beta, EDNRB |
| Okino et al [43] | LNCaP, RWPE-1,  PWR-1E | 0.25 or 1µM; 5-aza-CdR  replenished on day 4 for 7 days OR,  1µM; 5-aza-CdR  replenished on day 3, 6 and 10 for 13 days | CYP1A1 |
| Das et al [44] | LNCaP | 1µM; 5-aza-CdR  one time treatment for 7 days | TMS1/ASC |
| Qin et al [45] | DU145, LNCaP, PC3 | 5µM; 5-aza-CdR  one time treatment for 5 days | WWOX |
| Shao et al [46] | DU145, LNCaP | 20µM; 5-aza-CdR  daily treatment for 4 days | Betaig-h3 |
| Fox et al [47] | DU145, PC3, PC3-ML (invasive PC3 subclone ) | 100µM; 5-aza-CdR  one time treatment for 3 days | Eph receptors and ephrin ligands |
| Sidiropoulos et al [48,49] | LNCaP, PC3 | 50 or 100µM; 5-aza-CdR  one time treatment for 2 days followed by drug-free media for 2 days OR,  1, 2, 3, 5 or 10µM; 5-aza-CdR  one time treatment for 5 days | Kallikrein genes |
| Chuang et al [50] | PC3 | 1µM; 5-aza-CdR  one time treatment for 24h followed by drug-free media for 5 days | P16, MAGE-A1, Alu and LINE1 repetitive elements |
| Kim et al [51] | DU145, LNCaP,  MDA-PCa-2a, PC3 | 1µM; 5-aza-CdR  one time treatment for 4-6 days OR,  1µM; 5-aza-CdR  every 4 days for 10 days | Identification of epigenetically-regulated candidate tumor suppressor genes (ALDH1a2) |
| Zhao et al [52] | DUPro, LNCaP, ND1, PC3 | 10µM; 5-aza-CdR  3 non-consecutive treatments on day 0, 2, 4. Cells grown in fresh media on day 1 and 3. Harvested on day 5 | TGF-beta II |
| Khorchide et al [53] | DU145, PNT-2 | 10µM; 5-aza-CdR  daily treatment for 5 days | CYP24, CYP27B1 |
| Enokida et al [54] | DU145, DUPro, LNCaP, ND1, PC3 | 5µM; 5-aza-CdR  one time treatment for 4 days | GSTP1 |
| Shiina et al [55] | DUPro, LNCaP, ND1, PC3 | 5µM; 5-aza-CdR  one time treatment for 4 days | Identification of epigenetically-regulated gamma-catenin genes |
| Mckie et al [56] | DU145, LNCaP, PC3-M | 2µM; 5-aza-CdR  3 non-consecutive treatments on day 0, 2 and 4. Cells were washed and grown in fresh media on day 1, 3 and harvested on day 5 | hSPRY2 |
| Yamada et al ^[57]^ | DU145, LNCaP | 1 or 5µM; 5-aza-CdR  one time treatment for 3 days | NTRK2 |
| Enokida et al ^[58]^ | DU145, DUPro, LNCaP, ND1 | 5µM; 5-aza-CdR  one time treatment for 4 days | MDR1 |
| Mutaguchi et al ^[59]^ | BPH-1, DU145, DUPro, LNCaP, ND1, PC3, TSU | 5µM; 5-aza-CdR  one time treatment for 4 days | IGFBP-rp1 |
| Gober et al ^[60]^ | PC3 | 2µM; 5-aza-CdR  one time treatment for 4 days | H11 |
| Liu et al ^[61]^ | LNCaP | 2, 5 or 10µM; 5-aza-CdR  one time treatment for 4 days | RASSF1A |
| Sasaki et al [62] | DU145, DUPro, ND1, LNCaP, PC3 | 8.8µM; 5-aza-CdR  new drug-containing media on day 2 and 4 for 5 days | ER, PR and AR |
| Lin et al [63] | LNCaP | 10µM; 5-aza-CdR  one time treatment for 7 or 14 days | GSTP1 |
| Lin et al [64] | LNCaP | 5µM; 5-aza-CdR  treatment regime not reported | GSTP1 |
| Sekita et al [65] | DU145, LNCaP, PC3, TSU-Pr1 | 0.5 or 2µM; 5-aza-CdR  one time treatment for 7 days | KAI1 |
| Patra et al [66] | BPH-1, DUPro, ND1, PC3, TSU-Pr1 | 10, 20, 35, 50, 75 or 100nM; 5-aza-CdR  one time treatment for 7 days | Effects on DNMTase activity |
| Singal et al [67] | LNCaP | 3, 5 or 10µM; 5-aza-CdR  daily treatment for 7 days | GSTP1 |
| Chlenski et al [68] | CA7T2CL, CWR22R, DU145, LNCaP, PC3 | 1µM ; 5-aza-CdR  one time treatment for 3 days | AR |
| Nakayama et al [69] | DU145, LNCaP, PC3 | 0.5 or 2µM; 5-aza-CdR  daily treatment for 3 days | AR |
| Usmani et al [70] | DU145, PC3 | 0.5µM; 5-aza-CdR  one time treatment for 24h | Neural endopeptidase (NEP) |
| Li et al [71] | BPH-1, DU145, DUPro, LNCaP, ND1, PC3, TSU-Pr1 | 8.8µM; 5-aza-CdR  3 non-consecutive treatments on day 0, 2 and 4. Cells grown in fresh media day 1 and 3; cells harvested day 5 | ER |
| Jarrard et al [72] | DU145, DUPro, LNCaP, PC3, PPC-1, TSU-Pr1 | 0.15 - 0.5µM; 5-aza-CdR  replenished every 2 days for 5 days | AR |
| Chi et al [73] | LNCaP, PC3, TSU-Pr1 | 0.5 or 1µM; 5-aza-CdR  one time treatment for 3- 6 days | P16 |
| Graff et al [74] | DU145, DUPro, FNC, LNCaP, PC3, TSU-Pr1 | 0.5µM; 5-aza-CdR  one time treatment for 3 days | E-cadherin |

**References**

1. Gravina GL, Festuccia C, Millimaggi D, Dolo V, Tombolini V, et al. (2008) Chronic azacitidine treatment results in differentiating effects, sensitizes against bicalutamide in androgen-independent prostate cancer cells. Prostate.

2. Shang D, Liu Y, Liu Q, Zhang F, Feng L, et al. (2009) Synergy of 5-aza-2'-deoxycytidine (DAC) and paclitaxel in both androgen-dependent and -independent prostate cancer cell lines. Cancer Lett 278: 82-87.

3. Walton TJ, Li G, Seth R, McArdle SE, Bishop MC, et al. (2008) DNA demethylation and histone deacetylation inhibition co-operate to re-express estrogen receptor beta and induce apoptosis in prostate cancer cell-lines. Prostate 68: 210-222.

4. Festuccia C, Gravina GL, D'Alessandro AM, Millimaggi D, Di Rocco C, et al. (2008) Downmodulation of dimethyl transferase activity enhances tumor necrosis factor-related apoptosis-inducing ligand-induced apoptosis in prostate cancer cells. Int J Oncol 33: 381-388.

5. Richter E, Masuda K, Cook C, Ehrich M, Tadese AY, et al. (2007) A role for DNA methylation in regulating the growth suppressor PMEPA1 gene in prostate cancer. Epigenetics 2: 100-109.

6. Bott SR, Arya M, Kirby RS, Williamson M (2005) p21WAF1/CIP1 gene is inactivated in metastatic prostatic cancer cell lines by promoter methylation. Prostate Cancer Prostatic Dis 8: 321-326.

7. Zhang Q, Rubenstein JN, Jang TL, Pins M, Javonovic B, et al. (2005) Insensitivity to transforming growth factor-beta results from promoter methylation of cognate receptors in human prostate cancer cells (LNCaP). Mol Endocrinol 19: 2390-2399.

8. Pulukuri SM, Rao JS (2005) Activation of p53/p21Waf1/Cip1 pathway by 5-aza-2'-deoxycytidine inhibits cell proliferation, induces pro-apoptotic genes and mitogen-activated protein kinases in human prostate cancer cells. Int J Oncol 26: 863-871.

9. Hurtubise A, Momparler RL (2004) Evaluation of antineoplastic action of 5-aza-2'-deoxycytidine (Dacogen) and docetaxel (Taxotere) on human breast, lung and prostate carcinoma cell lines. Anticancer Drugs 15: 161-167.

10. Kitagawa Y, Kyo S, Takakura M, Kanaya T, Koshida K, et al. (2000) Demethylating reagent 5-azacytidine inhibits telomerase activity in human prostate cancer cells through transcriptional repression of hTERT. Clin Cancer Res 6: 2868-2875.

11. Steiner MS, Wang Y, Zhang Y, Zhang X, Lu Y (2000) p16/MTS1/INK4A suppresses prostate cancer by both pRb dependent and independent pathways. Oncogene 19: 1297-1306.

12. Chang G, Xu S, Dhir R, Chandran U, O'Keefe DS, et al. Hypoexpression and Epigenetic Regulation of Candidate Tumor Suppressor Gene CADM-2 in Human Prostate Cancer. Clin Cancer Res 16: 5390-5401.

13. Ibragimova I, Ibanez de Caceres I, Hoffman AM, Potapova A, Dulaimi E, et al. Global reactivation of epigenetically silenced genes in prostate cancer. Cancer Prev Res (Phila Pa) 3: 1084-1092.

14. Kwabi-Addo B, Wang S, Chung W, Jelinek J, Patierno SR, et al. Identification of differentially methylated genes in normal prostate tissues from African American and Caucasian men. Clin Cancer Res 16: 3539-3547.

15. Muller I, Wischnewski F, Pantel K, Schwarzenbach H Promoter- and cell-specific epigenetic regulation of CD44, Cyclin D2, GLIPR1 and PTEN by methyl-CpG binding proteins and histone modifications. BMC Cancer 10: 297.

16. Song EY, Shurin MR, Tourkova IL, Gutkin DW, Shurin GV Epigenetic mechanisms of promigratory chemokine CXCL14 regulation in human prostate cancer cells. Cancer Res 70: 4394-4401.

17. Kwon O, Jeong SJ, Kim SO, He L, Lee HG, et al. Modulation of E-cadherin expression by K-Ras; involvement of DNA methyltransferase-3b. Carcinogenesis 31: 1194-1201.

18. Schayek H, Bentov I, Sun S, Plymate SR, Werner H Progression to metastatic stage in a cellular model of prostate cancer is associated with methylation of the androgen receptor gene and transcriptional suppression of the insulin-like growth factor-I receptor gene. Exp Cell Res.

19. Zhang P, Singh A, Yegnasubramanian S, Esopi D, Kombairaju P, et al. Loss of Kelch-like ECH-associated protein 1 function in prostate cancer cells causes chemoresistance and radioresistance and promotes tumor growth. Mol Cancer Ther 9: 336-346.

20. Wu M, Ho SM (2004) PMP24, a gene identified by MSRF, undergoes DNA hypermethylation-associated gene silencing during cancer progression in an LNCaP model. Oncogene 23: 250-259.

21. Zhang X, Wu M, Xiao H, Lee MT, Levin L, et al. Methylation of a single intronic CpG mediates expression silencing of the PMP24 gene in prostate cancer. Prostate 70: 765-776.

22. Majid S, Dar AA, Shahryari V, Hirata H, Ahmad A, et al. Genistein reverses hypermethylation and induces active histone modifications in tumor suppressor gene B-Cell translocation gene 3 in prostate cancer. Cancer 116: 66-76.

23. Pandey M, Shukla S, Gupta S (2009) Promoter demethylation and chromatin remodeling by green tea polyphenols leads to re-expression of GSTP1 in human prostate cancer cells. Int J Cancer.

24. Vanaja DK, Cheville JC, Iturria SJ, Young CY (2003) Transcriptional silencing of zinc finger protein 185 identified by expression profiling is associated with prostate cancer progression. Cancer Res 63: 3877-3882.

25. Vanaja DK, Ballman KV, Morlan BW, Cheville JC, Neumann RM, et al. (2006) PDLIM4 repression by hypermethylation as a potential biomarker for prostate cancer. Clin Cancer Res 12: 1128-1136.

26. He M, Vanaja DK, Karnes RJ, Young CY (2009) Epigenetic regulation of Myc on retinoic acid receptor beta and PDLIM4 in RWPE1 cells. Prostate 69: 1643-1650.

27. Cheng Y, Kim JW, Liu W, Dunn TA, Luo J, et al. (2009) Genetic and epigenetic inactivation of TNFRSF10C in human prostate cancer. Prostate 69: 327-335.

28. Guan M, Xu C, Zhang F, Ye C (2009) Aberrant methylation of EphA7 in human prostate cancer and its relation to clinicopathologic features. Int J Cancer 124: 88-94.

29. Liu Z, Marquez M, Nilsson S, Holmberg AR (2008) Incubation with somatostatin, 5-aza decitabine and trichostatin up-regulates somatostatin receptor expression in prostate cancer cells. Oncol Rep 20: 151-154.

30. Wang X, Tryndyak V, Apostolov EO, Yin X, Shah SV, et al. (2008) Sensitivity of human prostate cancer cells to chemotherapeutic drugs depends on EndoG expression regulated by promoter methylation. Cancer Lett 270: 132-143.

31. Liu JW, Nagpal JK, Sun W, Lee J, Kim MS, et al. (2008) ssDNA-binding protein 2 is frequently hypermethylated and suppresses cell growth in human prostate cancer. Clin Cancer Res 14: 3754-3760.

32. Chung W, Kwabi-Addo B, Ittmann M, Jelinek J, Shen L, et al. (2008) Identification of novel tumor markers in prostate, colon and breast cancer by unbiased methylation profiling. PLoS One 3: e2079.

33. Fujii S, Ito K, Ito Y, Ochiai A (2008) Enhancer of zeste homologue 2 (EZH2) down-regulates RUNX3 by increasing histone H3 methylation. J Biol Chem 283: 17324-17332.

34. Menschikowski M, Hagelgans A, Gussakovsky E, Kostka H, Paley EL, et al. (2008) Differential expression of secretory phospholipases A2 in normal and malignant prostate cell lines: regulation by cytokines, cell signaling pathways, and epigenetic mechanisms. Neoplasia 10: 279-286.

35. Cangemi R, Mensah A, Albertini V, Jain A, Mello-Grand M, et al. (2008) Reduced expression and tumor suppressor function of the ETS transcription factor ESE-3 in prostate cancer. Oncogene 27: 2877-2885.

36. Benbrahim-Tallaa L, Waterland RA, Dill AL, Webber MM, Waalkes MP (2007) Tumor suppressor gene inactivation during cadmium-induced malignant transformation of human prostate cells correlates with overexpression of de novo DNA methyltransferase. Environ Health Perspect 115: 1454-1459.

37. Dubovsky JA, McNeel DG (2007) Inducible expression of a prostate cancer-testis antigen, SSX-2, following treatment with a DNA methylation inhibitor. Prostate 67: 1781-1790.

38. Hoffmann MJ, Muller M, Engers R, Schulz WA (2006) Epigenetic control of CTCFL/BORIS and OCT4 expression in urogenital malignancies. Biochem Pharmacol 72: 1577-1588.

39. Wlazlinski A, Engers R, Hoffmann MJ, Hader C, Jung V, et al. (2007) Downregulation of several fibulin genes in prostate cancer. Prostate 67: 1770-1780.

40. Kawamoto K, Okino ST, Place RF, Urakami S, Hirata H, et al. (2007) Epigenetic modifications of RASSF1A gene through chromatin remodeling in prostate cancer. Clin Cancer Res 13: 2541-2548.

41. Hasegawa Y, Matsubara A, Teishima J, Seki M, Mita K, et al. (2007) DNA methylation of the RIZ1 gene is associated with nuclear accumulation of p53 in prostate cancer. Cancer Sci 98: 32-36.

42. Bastian PJ, Ellinger J, Heukamp LC, Kahl P, Muller SC, et al. (2007) Prognostic value of CpG island hypermethylation at PTGS2, RAR-beta, EDNRB, and other gene loci in patients undergoing radical prostatectomy. Eur Urol 51: 665-674; discussion 674.

43. Okino ST, Pookot D, Li LC, Zhao H, Urakami S, et al. (2006) Epigenetic inactivation of the dioxin-responsive cytochrome P4501A1 gene in human prostate cancer. Cancer Res 66: 7420-7428.

44. Das PM, Ramachandran K, Vanwert J, Ferdinand L, Gopisetty G, et al. (2006) Methylation mediated silencing of TMS1/ASC gene in prostate cancer. Mol Cancer 5: 28.

45. Qin HR, Iliopoulos D, Semba S, Fabbri M, Druck T, et al. (2006) A role for the WWOX gene in prostate cancer. Cancer Res 66: 6477-6481.

46. Shao G, Berenguer J, Borczuk AC, Powell CA, Hei TK, et al. (2006) Epigenetic inactivation of Betaig-h3 gene in human cancer cells. Cancer Res 66: 4566-4573.

47. Fox BP, Tabone CJ, Kandpal RP (2006) Potential clinical relevance of Eph receptors and ephrin ligands expressed in prostate carcinoma cell lines. Biochem Biophys Res Commun 342: 1263-1272.

48. Sidiropoulos M, Pampalakis G, Sotiropoulou G, Katsaros D, Diamandis EP (2005) Downregulation of human kallikrein 10 (KLK10/NES1) by CpG island hypermethylation in breast, ovarian and prostate cancers. Tumour Biol 26: 324-336.

49. Pampalakis G, Diamandis EP, Sotiropoulou G (2006) The epigenetic basis for the aberrant expression of kallikreins in human cancers. Biol Chem 387: 795-799.

50. Chuang JC, Yoo CB, Kwan JM, Li TW, Liang G, et al. (2005) Comparison of biological effects of non-nucleoside DNA methylation inhibitors versus 5-aza-2'-deoxycytidine. Mol Cancer Ther 4: 1515-1520.

51. Kim H, Lapointe J, Kaygusuz G, Ong DE, Li C, et al. (2005) The retinoic acid synthesis gene ALDH1a2 is a candidate tumor suppressor in prostate cancer. Cancer Res 65: 8118-8124.

52. Zhao H, Shiina H, Greene KL, Li LC, Tanaka Y, et al. (2005) CpG methylation at promoter site -140 inactivates TGFbeta2 receptor gene in prostate cancer. Cancer 104: 44-52.

53. Khorchide M, Lechner D, Cross HS (2005) Epigenetic regulation of vitamin D hydroxylase expression and activity in normal and malignant human prostate cells. J Steroid Biochem Mol Biol 93: 167-172.

54. Enokida H, Shiina H, Urakami S, Igawa M, Ogishima T, et al. (2005) Ethnic group-related differences in CpG hypermethylation of the GSTP1 gene promoter among African-American, Caucasian and Asian patients with prostate cancer. Int J Cancer 116: 174-181.

55. Shiina H, Breault JE, Basset WW, Enokida H, Urakami S, et al. (2005) Functional Loss of the gamma-catenin gene through epigenetic and genetic pathways in human prostate cancer. Cancer Res 65: 2130-2138.

56. McKie AB, Douglas DA, Olijslagers S, Graham J, Omar MM, et al. (2005) Epigenetic inactivation of the human sprouty2 (hSPRY2) homologue in prostate cancer. Oncogene 24: 2166-2174.

57. Yamada Y, Toyota M, Hirokawa Y, Suzuki H, Takagi A, et al. (2004) Identification of differentially methylated CpG islands in prostate cancer. Int J Cancer 112: 840-845.

58. Enokida H, Shiina H, Igawa M, Ogishima T, Kawakami T, et al. (2004) CpG hypermethylation of MDR1 gene contributes to the pathogenesis and progression of human prostate cancer. Cancer Res 64: 5956-5962.

59. Mutaguchi K, Yasumoto H, Mita K, Matsubara A, Shiina H, et al. (2003) Restoration of insulin-like growth factor binding protein-related protein 1 has a tumor-suppressive activity through induction of apoptosis in human prostate cancer. Cancer Res 63: 7717-7723.

60. Gober MD, Smith CC, Ueda K, Toretsky JA, Aurelian L (2003) Forced expression of the H11 heat shock protein can be regulated by DNA methylation and trigger apoptosis in human cells. J Biol Chem 278: 37600-37609.

61. Liu L, Yoon JH, Dammann R, Pfeifer GP (2002) Frequent hypermethylation of the RASSF1A gene in prostate cancer. Oncogene 21: 6835-6840.

62. Sasaki M, Tanaka Y, Perinchery G, Dharia A, Kotcherguina I, et al. (2002) Methylation and inactivation of estrogen, progesterone, and androgen receptors in prostate cancer. J Natl Cancer Inst 94: 384-390.

63. Lin X, Asgari K, Putzi MJ, Gage WR, Yu X, et al. (2001) Reversal of GSTP1 CpG island hypermethylation and reactivation of pi-class glutathione S-transferase (GSTP1) expression in human prostate cancer cells by treatment with procainamide. Cancer Res 61: 8611-8616.

64. Lin X, Tascilar M, Lee WH, Vles WJ, Lee BH, et al. (2001) GSTP1 CpG island hypermethylation is responsible for the absence of GSTP1 expression in human prostate cancer cells. Am J Pathol 159: 1815-1826.

65. Sekita N, Suzuki H, Ichikawa T, Kito H, Akakura K, et al. (2001) Epigenetic regulation of the KAI1 metastasis suppressor gene in human prostate cancer cell lines. Jpn J Cancer Res 92: 947-951.

66. Patra SK, Patra A, Dahiya R (2001) Histone deacetylase and DNA methyltransferase in human prostate cancer. Biochem Biophys Res Commun 287: 705-713.

67. Singal R, van Wert J, Bashambu M (2001) Cytosine methylation represses glutathione S-transferase P1 (GSTP1) gene expression in human prostate cancer cells. Cancer Res 61: 4820-4826.

68. Chlenski A, Nakashiro K, Ketels KV, Korovaitseva GI, Oyasu R (2001) Androgen receptor expression in androgen-independent prostate cancer cell lines. Prostate 47: 66-75.

69. Nakayama T, Watanabe M, Suzuki H, Toyota M, Sekita N, et al. (2000) Epigenetic regulation of androgen receptor gene expression in human prostate cancers. Lab Invest 80: 1789-1796.

70. Usmani BA, Shen R, Janeczko M, Papandreou CN, Lee WH, et al. (2000) Methylation of the neutral endopeptidase gene promoter in human prostate cancers. Clin Cancer Res 6: 1664-1670.

71. Li LC, Chui R, Nakajima K, Oh BR, Au HC, et al. (2000) Frequent methylation of estrogen receptor in prostate cancer: correlation with tumor progression. Cancer Res 60: 702-706.

72. Jarrard DF, Kinoshita H, Shi Y, Sandefur C, Hoff D, et al. (1998) Methylation of the androgen receptor promoter CpG island is associated with loss of androgen receptor expression in prostate cancer cells. Cancer Res 58: 5310-5314.

73. Chi SG, deVere White RW, Muenzer JT, Gumerlock PH (1997) Frequent alteration of CDKN2 (p16(INK4A)/MTS1) expression in human primary prostate carcinomas. Clin Cancer Res 3: 1889-1897.

74. Graff JR, Herman JG, Lapidus RG, Chopra H, Xu R, et al. (1995) E-cadherin expression is silenced by DNA hypermethylation in human breast and prostate carcinomas. Cancer Res 55: 5195-5199.
